# Understanding nutrition challenges and information needs of women undergoing cytoreductive surgery for ovarian cancer: a study protocol for an experience-based co-design methodology

**DOI:** 10.1007/s00520-026-10318-5

**Published:** 2026-01-15

**Authors:** Kathryn Cherry, Emma Quigley, Cherry Koh, Aisling Scully, Rhonda Farrell, Adrienne Young, Margaret Allman-Farinelli, Sharon Carey

**Affiliations:** 1 Faculty of Medicine and Health, The University of Sydney, Sydney, NSW Australia; 2 Nutrition and Dietetics Department, Royal Prince Alfred Hospital, Sydney, NSW Australia; 3 Consumer Representative, Sydney, NSW Australia; 4 Institute of Academic Surgery, Royal Prince Alfred Hospital, Sydney, NSW Australia; 5 Chris O’Brien Lifehouse, Sydney, NSW Australia; 6 Dietetics and Food Services, Royal Brisbane and Women’s Hospital, Brisbane, QLD Australia; 7 Centre for Health Services Research, The University of Queensland, Brisbane, QLD Australia

**Keywords:** Ovarian cancer, Malnutrition, Surgery, Co-design, Patient involvement, Surgical menopause

## Abstract

**Purpose:**

Cytoreductive surgery (CRS) with or without intraperitoneal hyperthermic chemotherapy (HIPEC) is a complex surgery aimed at removing peritoneal surface malignancy (PSM). PSM is prevalent in ovarian cancer, with up to 85% of patients experiencing non-specific symptoms leading to malnutrition. Studies have shown oncology patients display confusion about nutrition recommendations, although understudied in ovarian cancer. Experience-based co-design is a method of participatory research with key principles including empowerment and collaboration. This project aims to utilise experience-based co-design to understand nutrition challenges and develop nutrition resources for women undergoing CRS ± HIPEC for ovarian cancer. The secondary aim is to evaluate the experience-based co-design process to ensure it aligns with key principles of co-design.

**Methods:**

This multi-centre study utilises experience-based co-design. Using maximum variation sampling, women with lived experience of CRS ± HIPEC for ovarian cancer, along with experienced oncology healthcare professionals will be invited to participate. Participants will be interviewed to identify key nutrition issues and information needs. Using thematic analysis, a patient journey map will be developed and key themes identified. A feedback session will be held for participants to identify priorities for service improvement and patient resource development based on key issues identified by patient journey mapping. These priorities will be addressed in co-design workshops with participants with lived experience and clinicians to develop patient information resources. Resources and experience-based co-design processes will be evaluated at the completion of the workshops.

**Conclusions:**

This study will deliver new insights into the nutrition challenges and information needs identified by people undergoing CRS ± HIPEC for ovarian cancer within a co-design approach.

## Introduction

Globally, ovarian cancer was the third most common gynaecological cancer in 2020, with a rise in incidence among younger women [1]. Due to the non-specified symptoms, diagnosis often occurs in advanced stages for 70% of cases [2], leading to poorer prognosis. The recurrence rate in advanced disease is 80%, compared with 15% in early ovarian cancer diagnoses [3]. Malignant metastases to the peritoneal surfaces are prevalent in 60–70% of patients diagnosed with a primary epithelial ovarian cancer, compared to < 10% of all other gynaecological cancers [2].

Cytoreductive surgery (CRS) with or without hyperthermic intraperitoneal chemotherapy (HIPEC) has been instrumental in improving survival outcomes in this population [2–4]. CRS is a complex surgical procedure with the goal of complete tumour resection and removal of all macroscopic disease. It involves removal of the ovaries, uterus and omentum and may involve resections of the bowel, liver or lymph nodes and peritoneal stripping [5]. A study reporting on patient experiences after CRS ± HIPEC found that at 6–12 months post-operatively, 35% still experienced bowel changes and 25% remained on a restrictive diet [6]. However, this study did not include ovarian primary, limiting the generalisability in this cohort.

At the time of ovarian cancer diagnosis, up to 85% of women may experience abdominal distention, fatigue, nausea, anorexia, weight loss, and/or constipation [2]. Ovarian cancer has a substantial impact on nutrition status, with up to 70% of women presenting with malnutrition, assessed using a validated assessment tool [7, 8]. Malnutrition has been associated with post-operative complications [9, 10] and adverse effects on overall survival in ovarian cancer [7, 11]. Despite this, there is currently a lack of good quality studies evaluating nutrition interventions specific to ovarian cancer [11].

In pre- and peri-menopausal women, removal of the ovaries during CRS ± HIPEC will induce surgical menopause. Due to the rapid change in hormone levels, rapid onset of more severe menopausal symptoms occurs compared to natural menopause [12]. This has been shown to negatively impact health related quality-of-life [13]. An Australian study assessing the presence of vasomotor symptoms and quality of life in peri-menopausal women undergoing risk-reducing salpingo-oophorectomy found that at 3 months post-surgery > 50% of women experienced hot flashes versus 10% in the comparison group [13].

Long term hypoestrogenism (upwards of 10 years) is associated with metabolic changes including increased intra-abdominal adiposity and waist circumference without significant change in weight or physical activity, along with increased total cholesterol levels [14]. This places pre- and peri-menopausal women at risk of metabolic changes including bone loss and cardiovascular disease. When not contraindicated, guidelines recommend commencement of hormone replacement therapy and dietary counselling to minimise the effects of long term hypoestrogenism and severity of vasomotor symptoms [15]. Despite this, study’s report a broad range of hormone replacement therapy uptake (6–82%) [16]. It has been shown that women experiencing surgical menopause may have immediate difficulty in retaining verbal information compared to women undergoing natural menopause [12]. This has implications for cognitive ability to understand and action medical information.

It is common for people diagnosed with cancer to seek alternative therapies and access unverified forms of nutrition information. In recent years, with the increase in patient-centred research, evidence is emerging that patients want more information than the public health system is currently offering [17–19]. A recent study in Australia showed 55% of people living with cancer turn to the internet for nutrition information after treatment. Whilst those surveyed found the information easy to understand (89%), they also reported that the information available on the internet was conflicting (52%) [17]. Patients are often left confused about dietary recommendations throughout and after cancer treatment [17, 19].

Co-design is a collaborative approach to facilitate consumer engagement when reviewing healthcare services. It has a high level of consumer involvement, exceeding the norms of ‘inform, consult, involve’ within the level of public participation spectrum [20] and observes co-design reaching ‘collaborate and empower’. This level of participation sees consumers making and leading decisions in relation to the research design and methodology, with a promise to implement what the consumers have decided [20]. By involving and partnering with consumers at this higher level, there is the potential for health services to create patient-centred care directly aligned with consumer needs.

Co-design has many definitions [21], a body of work by Young and Christoffsen 2022 defined co-design as ‘a process where people with professional and lived experience partner as equals to improve health services by listening, learning and making decisions together’ [22]. One such co-design methodology is ‘experience-based co-design’ which follows 8 steps, outlined in Table 1 [23]. Whilst there is an overall lack of nutrition-focused co-design studies [24], an Australian study found a high level of acceptability of co-designed oncology nutrition resources in consumers, carers, and healthcare professionals [25].

**Table 1:** Description of the experience-based co-design steps adapted from the point of care foundation [23]

Step	Description
1	Observe the clinical area
2	Interview patients, staff and carers
3	Identify key themes and develop engaging film
4	Service provider feedback event
5	Service user feedback event
6	Joint event to review key themes and develop priorities
7	Co-design workshops to address key themes
8	Celebration event

A key component of experience-based co-design is the use of experiential learning such as patient journey mapping, to identify areas for service improvement [26]. Similar to co-design, there are many definitions of patient journey mapping. For the purposes of this study, Davies et al. (2022, p. 84) definition is utilised: ‘a patient-oriented project that has been undertaken to better understand barriers, facilitators, experiences, interactions with services and/or outcomes for individuals and/or their carer’s and family members as they navigate, experience and exit one or more services in a health system by documenting elements of the journey to produce a visual or descriptive map’ [26]. The scoping review details the main purposes for undertaking patient journey mapping in healthcare which included identifying service gaps and unmet needs and understanding the patient experience. It was common for a study to encompass more than one purpose [26].

There is a lack of nutrition-focused research specific to ovarian cancer and cytoreductive surgery. Hence, the primary aim of this study is to utilise the experience-based co-design methodology to understand and address the nutrition challenges and information needs of women diagnosed with ovarian cancer having undergone CRS ± HIPEC. The secondary aim is to evaluate the experience-based co-design process to ensure it aligns with the key principles of co-design.

## Materials and methods

### Study governance

A steering committee was formed which included a senior dietitian (KC) undertaking a higher degree by research, a leading gynaeoncological surgeon (RF), a leading colorectal and peritoneal malignancy surgeon (CK), a person with lived experience of ovarian cancer and the service (EQ), a Professor of Dietetics (MAF), and a senior dietitian and researcher (SC). The lead researcher (KC) received funding to undertake a higher research degree for 2 years. The funding will also be utilised to pay for patient involvement as per the ‘consumer, carer and community member remuneration’ guideline [27]. Ethics has been granted (2023/ETH01096, 2024/ETH01598, 2024/ETH01948) from the Sydney Local Health District Ethics Committee. This research will be undertaken in accordance with the Declaration of Helsinki principles.

### Lived experience co-design

A person with lived experience of ovarian cancer and the service has been engaged to sit on the steering committee as a consumer representative and co-researcher (EQ). They will contribute to defining the eligibility criteria, recruitment strategy, the data collection tools and interview guides, and will be involved in workshop design ± facilitation, data analysis, and report writing.

Regular debriefing will occur throughout the project, and referral to psychological support will be offered where distress is detected. They will be remunerated for meetings and workshops attended.

### Study design

**Fig. 1:**
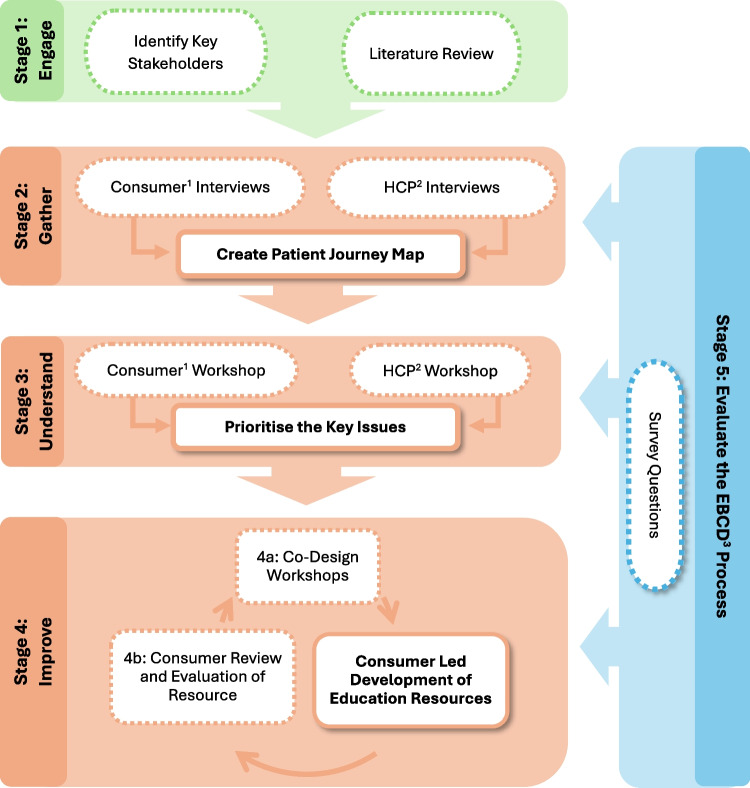
The five stages of the study aligned with the experience-based co-design methodology. ^1^Consumers refer to women with lived experience of ovarian cancer; ^2^HCP = Healthcare professionals

This is a multi-centre study across two tertiary referral hospitals in Sydney, Australia. Usual dietetic care differs slightly across sites. One hospital provides blanket pre-operative nutrition assessment, blanket inpatient nutrition care, and ad hoc post-discharge care as clinically required. The second site provides ad hoc pre-op nutrition assessments based on nutrition screening referrals, blanket inpatient nutrition assessments for women who underwent a bowel resection as part of their CRS and ad hoc post-discharge nutrition support as clinically appropriate. This is a co-design study based on the experience-based co-design framework [23] utilising qualitative interviews and co-design workshops. It involves the engagement of people with lived experience of ovarian cancer requiring CRS ± HIPEC in partnership with healthcare professionals with experience of the service to identify and prioritise key nutrition issues and co-design and evaluate a solution. Figure 1 depicts the 5 stages of this project and alignment with the experience-based co-design methodology.

### Participants

Women with lived experience will be recruited from two participating high-volume (> 50 cases per year) tertiary centres who are deemed well enough to participate by their treating surgeon. Inclusion criteria is as follows:

- Women who have undergone CRS ± HIPEC for advanced primary epithelial ovarian cancer in the preceding 18 months from time of ethics approval
- 18 years of age or older
- Have adequate English and cognition to consent and participate
- Have access to and ability to use technology to participate in virtual activities.

Formal consent will be obtained from a member of the research team who has not been involved in the patients’ care. Maximal variation sampling will be used to ensure a diverse range of participants representing various ages, geographical locations, and cultural backgrounds. Regular debriefing with participants will occur throughout the project, and referral to psychological support will be offered where distress is detected. Where participants with lived experience are required to attend online workshops, they will be remunerated for their time.

Healthcare professionals including surgeons, dietitians and clinical nurse consultants that have worked within the area of gynaecological surgery at either site in the previous 24 months from time of ethics approval will be invited to participate. Participants will need to have 6 month experience in the clinical area of surgical oncology. Formal consent will be obtained from a member of the research team who does not work directly with the potential participant. Maximal variation sampling will be used to ensure recruitment of healthcare professionals from different professions and with varying years of experience. Healthcare professionals will not be remunerated for their time.

### Procedure, data collection and analysis

#### Stage 1: engage

Observations of the peritoneal and gynae-oncological service will occur to gain an understanding of what patients and staff may experience (experience-based co-design step 1). The lead researcher will observe the pre-admission clinic, post-operative consultations with clinical nurse consultants, dietetic service pre- and post-operatively, and pre- and post-operative clinical consultations with the surgeons. These observations will assist in the development of the patient journey interview questions used in Stage 2.

#### Stage 2: gather

Semi-structured interviews will be conducted to gather the lived experience of patients and healthcare professionals’ perception of the patients’ journey. The interviews will have an emphasis on nutrition impact symptoms and patient information needs. It is expected this will require 15–20 women with lived experience and 5–10 healthcare professionals (experience-based co-design step 2). The number of health professionals is limited due to the specialist nature of this clinical area; it is the aim to recruit at least one health professional from each clinical area across both sites. The interview questions will be designed based on a review of the literature and feedback from people with lived experience. Questions will be piloted on two patients undergoing a similar surgery for a different primary tumor. A resource with various emoji faces depicting different emotions will be used throughout the interviews to assist in developing a visual and emotive patient journey map, adopted from the Agency for Clinical Innovation ‘Co-Design Toolkit’ [28].

Interviews will be approximately 60-min and conducted via Microsoft Teams to allow rural and regional participation. The interview will be conducted by a member of the research team (KC or AS) who has not been involved in the patients’ care or has not worked directly with the healthcare professional. KC and AS are both female senior clinical dietitians with experience working in surgical oncology.

Transcripts will be cleaned and de-identified by KC and AS. NVivo, version 14 (QRS International) will be used for data analysis. De-identified transcripts will be independently coded by two coders (KC and SC) to ensure credibility and consistency of the results. A subset of transcripts will be coded and reviewed by EQ to ensure triangulation of themes. Using thematic analysis [29], key themes will be identified.

A patient journey map will be developed depicting a visual representation of the patient and staff journey. Findings from this study will be submitted for publication and reported on using the COnsolidated criteria for REporting Qualitative research (COREQ) [30] reporting guideline and Guidance for Reporting Involvement of Patients and the Public (GRIPP2) reporting checklist [31].

#### Stage 3: understand

Two focus groups will take place via videoconferencing using Microsoft Teams (version 1416/1.0.0.2024203702) with an associated whiteboard application, ‘Mentimeter’ (version 2.5). One focus group will be attended by people with lived experience (*n* = 10–15), the other by healthcare professionals (*n* = 5–10). The key themes identified from Stage 2, along with the patient journey map, will be presented including de-identified quotes. Participants will be asked to rank and reach a consensus on the top nutrition priorities and information needs at each stage of the patient journey (experience-based co-design step 4–5).

Using the modified nominal group technique (NGT) outlined in Table 2 [32], participants will be asked to rank the key outcomes from highest to lowest priority. Step 2 and 3 of the NGT will be repeated up to three times until a consensus is reached [32]. The voting and ranking will be done anonymously via ‘Mentimeter’.

**Table 2:** An outline of the modified nominal group technique

Step	Description
1	Presentation of the key outcomes generated in stage 2
2	Break into small groups to discuss and clarify outcomes
3	Voting: each participant anonymously ranks each of the outcomes

On completion of the workshop, participants will be asked four questions to aid in developing the evaluation guide for the co-designed products in Stage 4 including (i) What would the nutrition resources look like?, (ii) What would it feel like to use the resources?, (iii) What would the data tell us?, and (iv) What would people be saying about the resources developed?. These questions have been adapted from the Metro North Queensland Co-Design toolkit [22].

In the event lived experience and healthcare professional focus groups result in different rankings, a third focus group will be held to reach a consensus between participants with lived experience and healthcare professionals. The third focus group will consist of a combination of participants with lived experience (*n* = 2–3) and HCPs (*n* = 1–2). The modified NGT will be repeated to reach a final ranking of the key outcomes (experience-based co-design step 6).

The results from Stage 3 will be used to inform Stage 4 of the project. Descriptive statistics of the group characteristics will be collated. The study will be submitted for consideration of publication and reported using the Standards for QUality Improvement Reporting Excellence (SQUIRE 2.0) [33] and GRIPP2 reporting checklist [31].

### Stage 4: improve

#### Stage 4a

Up to six 1-h co-design workshops will take place over a 6–9-month period (experience-based co-design step 7). The objective is to co-create a solution for women undergoing CRS ± HIPEC for ovarian cancer. To reduce the risk of power imbalance, the workshops will include predominantly women with lived experience (*n* = 3–5), while healthcare professionals (*n* = 1–2) will be invited to attend 25–50% of the workshops. The healthcare professionals, including a dietitian, will ensure the end product remains aligned with evidence-based practice. The workshops will be designed by KC and EQ under the guidance of AY, an experienced co-design researcher and facilitator. KC will attend training on designing and facilitating workshops.

These workshops will be held via Microsoft Teams with the use of a whiteboard application, ‘Mentimeter’. They will be led by an external facilitator to ensure objectivity, under the guidance of SC and AY, both experienced facilitators. As experience-based co-design is an iterative approach, the content of the workshops will be based on the key areas identified by participants as workshops progress. The first workshop will have predefined activities: establishing roles, responsibilities, and expectations; ensuring confidentiality; clarification of aims and processes; and reviewing the prioritised key outcomes from Stage 3. Subsequent sessions will be utilised to:

1. Review currently available resources
2. Generate ideas to meet information needs
3. To develop and review prototype resources.

#### Stage 4b

The newly developed resources will be tested prospectively with five patients from the health service. Women scheduled for CRS ± HIPEC with primary ovarian cancer, or who have had the surgery within the previous 3 months will be invited to participate. Participants will be asked to review the co-designed resources and complete a pre/post-questionnaire. Pre- and post-questionnaire data will be analysed using descriptive statistics and presented back to the co-design group from Stage 4a for final amendments and implementation.

### Stage 5: evaluate

The final stage will be to evaluate the experience-based co-design process. At the end of each workshop in stage 3 and 4, participants will be asked three evaluation questions including:

1. Do you feel you were heard today, scored on a Likert scale from one to five
2. What worked well today?
3. What could we do differently next time to improve participation?

An attendance log will be kept including demographics (age, cultural background and geographical location) to assess if diversity within the group was maintained. A summary of consumer and health professional hours will be collated to inform the burden of the co-design process. At the completion of Stage 4, all participants from Stage 2–4 will be invited via email or post to participate in a final survey to evaluate their experience of the experience-based co-design process. Stage 4 and 5 will be reported on and submitted for consideration of publication following COREQ [30], SQUIRE 2.0 [33] and GRIPP2 [31] reporting standards.

At the conclusion of Stage 5, a celebratory event will be held (experience-based co-design step 8). All participants through each stage of the project including the healthcare team will be invited to attend. The final results will be disseminated at this time. A final publication containing the results of each stage will be submitted for consideration of publication and presented at research conferences.

## Discussion

Ovarian cancer causes non-specified symptoms which can lead to malnutrition. Malnutrition is known to result in poorer surgical and treatment outcomes. By utilising the experience-based co-design approach, women with lived experience can offer unique insights to the challenges faced while undergoing treatment with CRS ± HIPEC that otherwise may have been overlooked. Experience-based co-design gives women with lived experience a voice and may allow the development of more sustainable health care solutions. A recent review of nutrition intervention focused co-design research found minimal in the oncology setting and only involving people with breast cancer [24]. A 2024 study on oncological nutrition interventions provided only generalised nutrition advice to a wide range of cancers [25]. This is the first study to our knowledge which encompasses the experience-based co-design process in addressing nutrition concerns and information needs identified by women undergoing CRS ± HIPEC for ovarian cancer. This protocol could be generalised to other complex health conditions or specific cancers and to add to the limited literature currently available.

There are many strengths to this study, including the collaborative nature between people with lived experience and healthcare professionals which will aid in developing resources that benefit both groups. By conducting the study online, it allows for women who live in remote areas to provide feedback and be involved in co-designing the solution. This study will contribute significant insight into the impact of surgical menopause and what information would be useful.

The authors acknowledge the limitations of this study. The availability of resourcing within public health may limit the time and buy-in from clinicians. This may pose a risk of truly adopting the experience-based co-design principles and authentic co-design. This study has been funded which allows payment of participants for their time and we acknowledge that this is not always possible in public health with its limited funding. This may impact the generalisability and feasibility of replicating the co-design methods in this study. Recruitment in a population who have a high recurrence rate and are undergoing treatment may create a challenge to the qualitative nature of this study. This is particularly true where cognition may be hindered.

## Conclusion

This body of work will assist in aligning the consumer needs and wants with evidenced based and patient-centred healthcare. It will aid in filling the current gap in literature surrounding the patient’s nutrition needs whilst undergoing surgical treatment for ovarian cancer. Valuable insights will be gained into the use of experience-based co-design and patient journey mapping in complex public healthcare and research.

## Data Availability

No new data were created or analyzed in this study. Data sharing is not applicable to this article.

## Abbreviations

*CRS*: Cytoreductive surgery
*HIPEC*: Hyperthermic intraperitoneal chemotherapy
*NGT*: Nominal group technique
*IAP2*: International association for public participation
*COREQ*: Consolidated criteria for reporting qualitative research
*SQUIRE*: Standard for quality improvement reporting excellence
*GRIPP2*: Guidance for reporting involvement of patients and the public

## References

[1] Huang J, Chan WC, Ngai CH et al (2022) Worldwide burden, risk factors, and temporal trends of ovarian cancer: a global study. Cancers (Basel) 14. 10.3390/cancers14092230 10.3390/cancers14092230 PMC9102475 35565359

[2] Cortés-Guiral D, Hübner M, Alyami M et al (2021) Primary and metastatic peritoneal surface malignancies. Nat Rev Dis Primers 7:1–23. 10.1038/s41572-021-00326-6 34916522 10.1038/s41572-021-00326-6

[3] de Bree E, Michelakis D, Anagnostopoulou E (2022) The current role of secondary cytoreductive surgery for recurrent ovarian cancer. Front. Oncol 12:1–12. 10.3389/fonc.2022.1029976 10.3389/fonc.2022.1029976 PMC9633943 36338689

[4] Kim SI, Kim JW (2021) Role of surgery and hyperthermic intraperitoneal chemotherapy in ovarian cancer. ESMO Open 6:1–9. 10.1016/j.esmoop.2021.100149 10.1016/j.esmoop.2021.100149 PMC8314869 33984680

[5] Lof P, van Soolingen NJ, Piek JMJ et al (2024) Preferences and considerations for interval cytoreductive surgery in advanced ovarian cancer: the patient’s perspective. Gynecol Oncol 187:227–234. 10.1016/j.ygyno.2024.05.018 38823307 10.1016/j.ygyno.2024.05.018

[6] Francescutti VA, Maciver AH, Stewart E et al (2019) Characterizing the patient experience of CS/HIPEC through in-depth interviews with patients: identification of key concepts in the development of a patient-centered program. Ann Surg Oncol 26:1063–1070. 10.1245/s10434-018-07120-x 30603814 10.1245/s10434-018-07120-x

[7] McQuellon R, Gavazzi C, Piso P, Swain D, Levine E (2008) Quality of life and nutritional assessment in peritoneal surface malignancy (PSM): recommendations for care. J Surg Oncol 98:300–305. 10.1002/jso.21050 18726903 10.1002/jso.21050

[8] Laky B, Janda M, Bauer J, Vavra C, Cleghorn G, Obermair A (2007) Malnutrition among gynaecological cancer patients. Eur J Clin Nutr 61:642–646. 10.1038/sj.ejcn.1602540 17021596 10.1038/sj.ejcn.1602540

[9] Reece L, Dragicevich H, Lewis C et al (2019) Preoperative nutrition status and postoperative outcomes in patients undergoing cytoreductive surgery and hyperthermic intraperitoneal chemotherapy. Ann Surg Oncol 26:2622–2630. 10.1245/s10434-019-07415-7 31123932 10.1245/s10434-019-07415-7

[10] Hübner M, Kusamura S, Villeneuve L et al (2020) Guidelines for perioperative care in cytoreductive surgery (CRS) with or without hyperthermic IntraPEritoneal chemotherapy (HIPEC): enhanced recovery after surgery (ERAS®) Society Recommendations - Part I: preoperative and intraoperative management. Eur J Surg Oncol 46:2292–2310. 10.1016/j.ejso.2020.07.041 32873454 10.1016/j.ejso.2020.07.041

[11] Benna-Doyle S, Baguley BJ, Laing E, Kiss N (2024) Nutritional interventions during treatment for ovarian cancer: a narrative review and recommendations for future research. Maturitas 183:107938. 10.1016/j.maturitas.2024.107938 38367367 10.1016/j.maturitas.2024.107938

[12] Kingsberg SA, Larkin LC, Liu JH (2020) Clinical effects of early or surgical menopause. Obstet Gynecol 135:853–868. 10.1097/AOG.0000000000003729 32168205 10.1097/AOG.0000000000003729

[13] Hickey M, Moss KM, Krejany EO et al (2021) What happens after menopause? (WHAM): a prospective controlled study of vasomotor symptoms and menopause-related quality of life 12 months after premenopausal risk-reducing salpingo-oophorectomy. Gynecol Oncol 163:148–154. 10.1016/j.ygyno.2021.07.029 34312002 10.1016/j.ygyno.2021.07.029

[14] Franklin RM, Ploutz-Snyder L, Kanaley JA (2009) Longitudinal changes in abdominal fat distribution with menopause. Metabolism 58:311–315. 10.1016/j.metabol.2008.09.030 19217444 10.1016/j.metabol.2008.09.030

[15] Nebgen DR, Domchek SM, Kotsopoulos J et al (2023) Care after premenopausal risk-reducing salpingo-oophorectomy in high-risk women: scoping review and international consensus recommendations. BJOG 130:1437–1450. 10.1111/1471-0528.17511 37132126 10.1111/1471-0528.17511 PMC7617419

[16] Armon S, Miron-Shatz T, Mor P et al (2023) BRCA carriers after risk-reducing bilateral salpingo-oophorectomy: menopausal hormone therapy knowledge gaps, and the impact of physicians’ recommendations. Climacteric 26:154–160. 10.1080/13697137.2023.2173567 36866779 10.1080/13697137.2023.2173567

[17] Baguley BJ, Benna-Doyle S, Drake S et al (2024) Access to nutrition services and information after active cancer treatment: a mixed methods study. J Cancer Surviv 18:176–185. 10.1007/s11764-023-01352-x 36823493 10.1007/s11764-023-01352-x PMC10866769

[18] Loeliger J, Dewar S, Kiss N, Drosdowsky A, Stewart J (2021) Patient and carer experiences of nutrition in cancer care: a mixed-methods study. Support Care Cancer 29:5475–5485. 10.1007/s00520-021-06111-1 33710413 10.1007/s00520-021-06111-1

[19] Keaver L, O’Callaghan N, Douglas P (2023) Nutrition support and intervention preferences of cancer survivors. J Hum Nutr Diet 36:526–539. 10.1111/jhn.13058 35778782 10.1111/jhn.13058

[20] International Association for Public Participation (2018) IAP2 public participation spectrum. [Online]. Available: https://iap2.org.au/resources/spectrum/. Accessed 07 March 2025

[21] Masterson D, Areskoug Josefsson K, Robert G, Nylander E, Kjellström S (2022) Mapping definitions of co-production and co-design in health and social care: a systematic scoping review providing lessons for the future. Health Expect 25:902–913. 10.1111/hex.13470 35322510 10.1111/hex.13470 PMC9122425

[22] Young A, Christoffersen A (n.d.) Co-design in metro north health [Online]. Available: https://metronorth.health.qld.gov.au/get-involved/co-design. Accessed 10 February 2025

[23] The Point of Care Foundation (n.d.) EBCD: evidence based co-design toolkit [Online]. Available: https://www.pointofcarefoundation.org.uk/resource/experience-based-co-design-ebcd-toolkit/. Accessed 29 November 2024

[24] Meloncelli N, Young A, Christoffersen A et al (2023) Co-designing nutrition interventions with consumers: a scoping review. J Hum Nutr Diet 36:1045–1067. 10.1111/jhn.13082 36056610 10.1111/jhn.13082

[25] Loeliger J, Francis J, Kiss N et al (2024) Enhancing the provision of cancer nutrition information to support care through experience-based co-design: a mixed-methods study. Support Care Cancer 32:257. 10.1007/s00520-024-08453-y 38556587 10.1007/s00520-024-08453-y

[26] Davies EL, Bulto LN, Walsh A et al (2023) Reporting and conducting patient journey mapping research in healthcare: a scoping review. J Adv Nurs 79:83–100. 10.1111/jan.15479 36330555 10.1111/jan.15479 PMC10099758

[27] NSW Health Patient Experience (2023) Consumer, carer and community member remuneration. NSW Government

[28] Agency for Clinical Innovation (n.d.) Co-design toolkit. NSW Government, [Online]. Available: https://aci.health.nsw.gov.au/projects/co-design. Accessed 07 March 2025

[29] Braun V, Clarke V (2006) Using thematic analysis in psychology. Qual Res Psychol 3:77–101. 10.1191/1478088706qp063oa

[30] Tong A, Sainsbury P, Craig J (2007) Consolidated criteria for reporting qualitative research (COREQ): a 32-item checklist for interviews and focus groups. Int J Qual Health Care 19:349–357. 10.1093/intqhc/mzm042 17872937 10.1093/intqhc/mzm042

[31] Staniszewska S, Brett J, Simera I et al (2017) GRIPP2 reporting checklists: tools to improve reporting of patient and public involvement in research. BMJ 358. 10.1136/bmj.j3453 10.1136/bmj.j3453 PMC5539518 28768629

[32] Dave MG, Chudyk AM, Oravec N et al (2022) Putting patient value first: using a modified nominal group technique for the implementation of enhanced recovery after cardiac surgery recommendations. JTCVS Open. 10.1016/j.xjon.2022.07.004 36590723 10.1016/j.xjon.2022.07.004 PMC9801247

[33] Ogrinc G, Davies L, Goodman D (2016) SQUIRE 2.0 (standards for quality improvement reporting excellence): revised publication guidelines from a detailed consensus process. BMJ Quality & Safety. 10.1016/j.xjon.2022.07.004 10.1136/bmjqs-2015-004411 PMC5256233 26369893

